# The Treatment of Varicocele, with a Clinical Report of Cases Treated by Amputation of the Scrotum

**Published:** 1883-10

**Authors:** G. Frank Lydston

**Affiliations:** Late Resident Surgeon, Charity and State Emigration Hospitals, New York. Lecturer on Genitourinary and Venereal Diseases, College of Physicians and Surgeons, Chicago, Ill.; 125 State St., Chicago


### ARTICLE II.

### THE TREATMENT OF VARICOCELE. WITH A CLINICAL REPORT OF CASES TREATED BY AMPUTATION OF THE SCROTUM.

By G. FRANK LYDSTON, M.D., Late Resident Surgeon, Charity and State Emigration Hospitals, New York. Lecturer on Genito-urinary and Venereal Diseases, College of Physicians and Surgeons, Chicago, Ill

After the elaborate monograph upon the subject of varicocele, written by Henry, there would seem to be but little to add to the subject, but having been an enthusiastic advocate of the operation of amputation of the redudant scrotum, and having had the opportunity of testing its efficacy I take pleasure in presenting some of my results and the conclusions drawn therefrom.

Varicocele, or varix, in its milder forms, at least, is not an affection which is intrinsically dangerous to life, but in nearly all well-marked cases it gives rise to considerable mental annoyance, as well as a varying degree of actual physical suffering. In nearly every young man who is affected with varicocele sufficiently marked to attract his attention, a greater or less degree of mental depression and sexual hypochondriasis exist, and, indeed, serve to make life miserable, in certain instances.

The physical deformity which varix may occasion may give rise to a great annoyance if the patient be at all sensitive, as is illustrated by Case V of the present series. In the slighter cases, such palliation as may be obtained by the use of a suspensory bandage and the application of cold and electricity may suffice to render the affection tolerable, and to prevent any increase in the size of the varix, but in the more severe cases the characteristic changes in the venous walls, due mainly to their loss of tone, and to connective tissue proliferation, go on, and we have as a result increased enlargement of the spermatic plexus, with all its attendant discomfort. In such instances the suspensory bandage fails to prevent a noticeable degree of deformity, and to allay the various symptoms completely. Its failure to relieve the patient's mental condition is especially evident, for the patient in removing and re-applying his bandage is made painfully cognizant of his deformity. The knowledge that he is unlike other young men, particularly as regards his sexual apparatus, has a peculiarly demoralizing effect upon such a patient unless he is unusually indifferent.

For such cases, then, we must devise some more effectual means of relief than are afforded by the ordinary measures of palliation. The various radical operations for varicocele are, as is well known, more or less dangerous to life, and to the functional integrity of the testicle of the affected side, and we ornarily prefer to avoid all risks, where practicable. To many surgeons the necessity of an operation for the relief of varicocele does not seem to be at all apparent, and, indeed, we must confess that the affection may be temporized with in a large number of cases. When, however, the patient suffers from the amount of mental and physical discomfort experienced by many of the cases that present themselves to us for treatment, something should be undertaken for their relief. An additional indication for operation lies in the danger of traumatic or spontaneous rupture of the diseased venous walls and the consequent formation of hæmatocele, which is, as is well known, a serious affection when involving the scrotum.

There is a popular idea that the danger of fatal hæmorrhage in operations for varicocele is very great, and as a consequence of this, and the tendency on the part of different surgeons to temporize with the affection, or turn the case over to instrument makers to have suspensories applied, the proportion of cases presenting themselves for operative treatment is very small. That a considerable degree of actual pain may be complained of in the more advanced cases, is exemplified by several of the cases described in the present paper.

I shall make no attempt to present the literature of varicocele, but will merely mention several of the operations suggested and practiced for its relief. We have a number of operations which have been recommended for the radical cure of varix; a radical cure implying the obliteration or inflammatory occlusion of the affected veins; among the most important of which are those of Vidal de Cassis, and Mr. Henry Lee. Vidal's method is familiar to most surgeons, and consists in passing an iron pin through the scrotum so as to pass between the vas deferens and the enlarged veins. A silver wire is then passed alongside the pin, in such a manner that the veins will be included between the pin and the wire, after which the wire is made fast to the pin at each end, and the pin twisted so as to bring a certain amount of com pression to bear upon the veins. This twisting is repeated every day or two, until the wire has ulcerated through the veins, after which the pin becomes loosened, and is removed. The veins are in this manner cut across, and occluded by inflammatory adhesion. Prof. T. M. Markoe has modified this operation by dispensing with the pin, and passing the silver wire in such a manner that it forms a loop, enclosing the veins, after which the ends of the loop are clamped to a lead plate upon the surface of the scrotum.

The wire is tightened from day to day, until the veins are severed, and the loop is free, when it is removed. Now, these operations, seemingly so simple and devoid of danger, are neither the one nor the other. Unless great caution be observed, hæmorrhage, with the formation of scrotal hæmatocele, may occur, from imperfect occlusion of the veins, and there is always more or less danger of exciting phlebitis. It is also by no means impossible for even surgeons of some experience to include the vas deferens in the loop of wire, an accident which will inevitably be followed by atrophy of the testicle, and may result in fatal tetanus as a result of peripheral irritation in this important structure. A case recently occurred in this city, in which a gentleman accidentally ligated the vas deferens in operating for varicocele, with the result of inducing atrophy of the testis. Atrophy of the testicle after the ligation of the spermatic veins, however, does not necessarily imply that the vas deferens has been included in the ligature, for such a result may ensue from simple interference with the nutrition of the organ consequent upon obstruction of the return circulation.

Mr. Henry Lee's operation is performed as follows: A segment of the anterior portion of the scrotum is excised, and the veins exposed, and temporarily compressed. The varicose vessels are then excised, and their cut extremities seared with the hot iron. When done antiseptically, this operation may yield excellent results, but we have still the same danger of atrophy of the testis that exists in the operations of Vidal and Markoe, although there is little danger of accidental deligation of the vas deferens, from the fact that the operation comprises exposure of the vessels instead of their ligation in the dark, if we may so express it. Now, it seems to me, that we are hardly warranted in performing operations of the nature of those described, until some safer method has been tried. In the amputation of the redundant scrotum, we have recourse to a measure which is far safer than the numerous radical operations, and, if properly performed, one which yields uniformly good results. Even in the event that in certain extreme cases we may be compelled to repeat the operation after the lapse of years, or even though the patient be compelled to wear a suspensory bandage for the remainder of his life, we will have answered all the indications in the case, and more especially the relief of the deformity and the sexual hypochondriasis. The Astley Cooper operation, then, so long neglected, is, as modified by Henry, a perfectly practicable means of relieving varicocele, and should be performed in preference to all other operations for this purpose. The fear of hæmorrhage excited in the minds of many, says Henry, "is now set aside by the perfect success following the operation."

Now, although, in my own experience, I have met with but one case in which apprehension, or even any great degree of annoyance, has been caused by hæmorrhage, and which in this case was due to a moderately well-marked hæmorrhagic diathesis, there has been a special tendency to venous oozing in my cases, which has led me to modify the operation somewhat, to obviate the disturbance of the wound, caused by the effused fluid. In the first two cases presented in this paper, I followed the details of the Henry operation in the main, using, however, his old form of clamp. In my later operations, I have modified the dressings somewhat, and, I believe, with advantage. Instead, of simple interrupted sutures of silk, I now use chromated catgut, for the interrupted sutures, with silver pins, and the figure of 8 silk suture, at the points of extreme tension, and at intervals varying with the amount of tension present or anticipated. Sutures of interrupted silk between the pins are not objectionable, but where convenient, I prefer catgut. The number of sutures should be sufficient to accurately approximate the cut surfaces. I find the silver pins much more trustworthy in cases characterized by extreme tension —and indeed, the tension is always great if a sufficient amount of scrotal tissue be removed—than any form of suture, whether of wire, silk or catgut.*

I find that the use of adhesive plaster in the first dressing, may advantageously be dispensed with. They are, however, of great service after the sutures and pins have been removed. As a substitute, I find graduated compresses to be all-sufficient in supporting the parts. These compresses may be made of carded

* I observe that Henry has recently advocated the use of silver pins also. I had used them prior to the publication of his remarks upon their advantages, and I believe they have been frequently used by other surgeons in the old method of operating.

oakum or borated cotton. After closing the wound I wash the surfaces with a warm saturated solution of boracic acid, and dust the edges of the wound with powdered iodoform. Prior to closing the lower angle of the wound I insert a drainage-tube of decalcified bone, which substance I much prefer to rubber. The wound having been properly cleansed and the edges approximated and sprinkled with iodoform, I place a piece of lint, in which holes are cut to allow the escape of discharges, smeared with carbolized vaseline, over the part, and then cover this with a piece of protective dipped in a 1-20 solution of carbolic acid. These dressings are for the purpose of preventing those which are placed above them from adhering to the part. Over the whole is placed a quantity of borated cotton, in such a manner that compression will be exerted upon the sides of the scrotum, thus supporting the wound. The dressings are retained in place by a large square of muslin, after the fashion of a diaper, a hole being cut for the penis. When the bowels move the bandage is removed, the dressings being supported by the hand of the patient while the bed-pan is being used.

I have been led to prefer the use of a warm solution of boracic acid to the ordinary cold solutions of carbolic acid, chiefly from the view that it is a more efficient antiseptic, is less likely to produce irritation, and is almost absolutely safe as far as the liability to injurious absorption is concerned. It is also of a temperature more nearly corresponding to the normal temperature of the tissues, and consequently is less likely to depress their vitality than are cold fluids. As a matter of simplicity, the dressing of iodoform and borated cotton is preferable to Lister's dressing, and as far as my own experience goes it is equally aseptic and antiseptic, and yields as uniformly good results.

It has been my experience that those cases in which healing by granulation rather than first intention, occurs, do better in the end, from the fact that the inflammatory thickening, and cicatricial contraction, yield a firmer support for the varix. A speedy union is, however, desirable, as the patient is usually anxious to get about, and the slower the method of healing, the greater the danger of inflammatory complications.

The most important point in the performance of amputation of the scrotum for varicocele is the amount of tissue to be removed. Henry's remarks upon this subject are so comprehensive that I take the liberty of repeating them: "Regarding the amount of scrotum to be removed, I can only say that I take away all that is not absolutely necessary to form a covering for the testicles. There is no danger of removing too much if the clamp be skillfully applied, and without skill there is more probability of a second operation being called for to remove more tissue." I can heartily endorse this statement of Henry's. I have observed that even in those cases in which the remaining portion of the scrotum is seemingly scanty at first, the tissues will soon accommodate themselves to the testes and yield all the room necessary.

I have mentioned the use of drainage tubes in the operation for varicocele, and I desire to state that from my experience in Cases I and V of this series I have been led to believe that drainage is an essential element of the operation, being indicated by the tendency to extreme tension from imprisoned blood. The primary hæmorrhage in these cases is usually slight, on account of the pressure of the clamp, but after this is removed and the wound has been closed there is a tendency to passive oozing of blood and serum, which may accumulate in considerable quantity, and even cause separation of the edges or giving way of the sutures. In Cases II, III and IV drainage tubes were used for forty-eight hours with the best results, union by first intention occurring, thus offering a marked contrast to those cases in which drainage tubes were not used. The same laxity or lack of tone of the vascular walls, which predisposes to varicocele principally,g ives risc to the passive oozingwhich causes the trouble in such cases.

This oozing is perhaps rarely if ever dangerous to life, but I think that it constitutes an important complication of the operation and one which should be guarded against. The drainage tube not only prevents the accumulation of blood, but it facilitates the application of styptics in case hæmorrhage should be excessive.

CASE I.—J. W., German, age 28; occupation, driver. This patient stated that he had been addicted to masturbation to an extreme degree until 22 years of age, at which time he married.

After marriage masturbation was substituted by sexual excesses. An enlargement of the left side of the scrotum was noticed at about the age of 20, and the patient was told at that time that he had a varicocele. During the last few years marked increase of the scrotal tumor had been evident, and a suspensory bandage had been worn for about a year. On admission to the hospital, the patient stated that his sexual powers were fast decreasing—probably as a consequence of mental depression as much as anything—and it was for this trouble chiefly that he applied for relief. Henry's modification of the Astley Cooper operation was performed, with, however, an old-style clamp. No drainage tubes were inserted, and the wound was closed partly with silk, and partly with silver wire. Secondary hæmorrhage occurred on the same day and was followed by some sloughing at the lower angle of the wound. Healing of this portion of the wound occurred quite rapidly, by granulation, the upper part uniting by first intention, and the wound being perfectly healed by twentieth day, at which time the patient was discharged. When last heard from, one year after the operation, he expressed himself as well pleased with the result of the operation, and entirely relieved of his symptoms.

CASE II.—Also a hospital case; a Bohemian about 23 or 24 years of age, and unable to speak English or German, and consequently to give any history of his trouble. The patient was debilitated, and appeared to suffer considerable annoyance from his varix, complaining of pain in the back and the affected testicle, as nearly as could be made out. The varicocele was very large, and involved the left spermatic plexus. The scrotal veins were greatly enlarged and varicose. After the administration of tonics and a nourishing diet for several weeks, the scrotum was amputated, as in the preceding case. There was very little hæmorrhage attendant upon the operation, and no secondary oozing of any consequence, but unfortunately, through carelessness of the nurse, erysipelas was set up in the wound on the third day, which resulted in considerable sloughing of the scrotal tissues, and prolonged healing for five weeks. At the end of this time, however, union was perfect. The result was all that could be desired, and the patient left the hospital in an apparently contented frame of mind. The subsequent history of this case, like the majority of hospital cases, could not be learned.

CASE III.—W. F., age 30; occupation, clerk. This gentleman stated that he had always been rather feeble, although enjoying fair health. Both masturbation and sexual excesses were acknowledged. Enlargement of the left side of the scrotum began to be noticeable at about the age of 18, and has since greatly increased, but had never been examined prior to his consulting me. By the advice of a friend he began wearing a suspensory bandage some years ago, and has used one more or less constantly ever since. Three years before I saw the case syphilis was contracted, and it was for its tertiary manifestations that I was consulted. On examination syphilitic nodes over both tibæ were found, and a history of the ordinary course of secondary syphilis was elicited. Severe osteocopic pains at night were complained of chiefly, and in addition great pain and discomfort from the varicocele were prominent sources of annoyance. A moderate varix of the right side was present, an occurrence not very frequently seen. Under the mixed treatment and a generous diet the condition of the patient rapidly improved, so that in four weeks I felt warranted in operating for the varicocele. In this case I modified the operation somewhat. Carbolized cat-gut was used instead of silk, and a drainage tube of decalcified bone inserted. As an additional support, four silver pins with the figure of 8 suture were inserted at the points of greatest tension. The wound was dressed with borated cotton and iodoform. Considerable venous oozing occurred in this case, but it was quite readily conducted away by the drainage tube, and produced no trouble whatever. Union by first intention was perfect throughout, and the drainage tube and pins were removed on the second and fourth days respectively, the sutures being allowed to soften and come away spontaneously. On the tenth day the patient was given a suspensory bandage, and allowed to go about and attend to his business. When last heard from (one year after the operation) he had dispensed with the suspensory bandage, and was feeling perfectly well. Small doses of hydrarg. had been taken meanwhile, and there had been no return of his syphilis.

CASE IV.—F. S., age 28; occupation, book-keeper. This patient stated that he had always been somewhat delicate, but had had little real sickness. Had never masturbated to any extent, nor been addicted to sexual excesses. Enlargement of the left side of the scrotum began at about the age of 21. Had had no treatment, nor had anything been done save the application of a rather rudely constructed suspensory bandage. On examination, a varix of moderate size was observed, and also a moderate varicosity of the veins of the extremities. The patient appears well in other respects, although evidently of not very robust physique, and seeks relief for so-called "spermatorrhœa," which, upon investigation, proved to consist of a nocturnal emission occurring once a fortnight. Considerable mental depression, with pain in the back, and testis were also complained of, the sense of painful, dragging weight upon the spermatic cord being especially annoying. The same operation was performed as in Case III., chromated gut, however, being substituted for the ordinary carbolized, and a hot saturated solution of boracic acid being used to wash the wounded surfaces. Five silver pins were inserted, and the iodoform and borated cotton dressing applied. Union was perfect throughout by first intention, the pins being removed on the third day. On the eleventh day a suspensory bandage was applied, and the patient allowed to resume business. Thirteen months after the operation this patient remained perfectly well, although by my advice he still wore a suspensory bandage.

CASE V.—W. H., age 21; occupation, amanuensis. This patient was always delicate, and is of a lymphatic temperament, although he has, as a rule, enjoyed comparatively good health. Has always been of a somewhat hæmorrhagic diathesis, and had consequent apprehension of hæmorrhage from an operation. About three years prior to his consulting me (August '81) this gentleman noticed an enlargement of the left side of the scrotum, from which, however, little trouble was experienced, save from mental disquiet, due to the knowledge of his deformity, until about a year ago, when he began to be annoyed by pain in the back and testis, sometimes radiating into the inner aspect of left thigh. These disturbances had gradually increased up to the date mentioned. When I examined this patient he stated that nothing had been done for the trouble, and that he was suffering quite severely from neuralgic pains in back, spermatic cord, and thighs, with a sense of dragging, increased on movement. Marked sexual hypochondriasis, with frequent nocturnal pollutions, were also prominent features of the case at this time. Neuralgia of the vesical neck, with frequent calls to urinate, also gave rise to great annoyance. The principal complaint on the part of this gentleman was that the deformity was very conspicuous, and it was mainly for this that he desired treatment. As his circumstances were not favorable to an operation, I followed a simple course of palliation, pending such a time as he should select for its performance. The nocturnal emissions and vesical neuralgia yielded readily to the cold sound. By these means and the application of a suspensory the patient was so far relieved that he deferred the operation for more than a year, but in March of present year decided to have it performed. I was unable to secure or prepare the chromic gut in this instance, and hence used carbolized silk. For the same reason drainage tubes were omitted. I found it necessary to remove a large segment of the scrotum, the varix being the largest I have ever seen. Five silver pins were inserted, and the boracic acid, borated cotton and iodoform dressing applied as usual. Venous oozing was not profuse, on account of the pressure of the clamp, during the operation. Union by first intention occurred throughout, but on the third day great pain and tension about the wound was experienced, and the lower portion of the wound was separated by clots contained in the scrotal cavity. On opening up the wound and removing the lower sutures and pins, I found a large amount of coagula—at least several ounces—and on turning these out quite a cavity was exposed. Venous oozing persisted for a week, when it ceased, and healthy granulations appeared. Healing was now quite rapid, and in a few days the wound was entirely closed, leaving an excellent result. The scrotum, at first rather tight, soon accommodated itself to the testes, and no trouble from this source was experienced. The relief afforded by the operation has been very marked, both mental and physical improvement being evident. The patient was attending to business on the twentieth day, and had drainage tubes been used this period might have been reduced one-half.

To secure a successful result from the operation of amputation of the redundant scrotum for varicocele, various general and local measures are indicated after the wound has healed, and these points are too often neglected. The most important point is the continuous wearing of a suspensory bandage for several years, or in certain instances, for life. This bandage should not only support, but should compress the parts, a large or ill-fitting bandage being useless. The patient should take a sponge bath daily, and should keep the bowels freely open, for, as is well known, the pressure of a distended colon upon the left spermatic vein has an important influence in the causation of varicocele. Daily applications of cold salt and water, and the Faradaic current, are useful. Internally, the mineral tonics, strychnia and ergot, should be given. These various measures tend, after a time, to restore the normal tone and resiliency of the venous walls, to a certain extent, and prevent a recurrence of the varicocele.

125 State St., Chicago, May 1, 1883.

